# Response: Commentary: Enhanced Monitoring of the Preterm Infant during Stabilization in the Delivery Room

**DOI:** 10.3389/fped.2016.00073

**Published:** 2016-07-18

**Authors:** Daragh Finn, Geraldine B. Boylan, C. Anthony Ryan, Eugene M. Dempsey

**Affiliations:** ^1^Department of Paediatrics and Child Health, University College Cork, Cork, Ireland; ^2^Irish Centre for Fetal and Neonatal Translational Research, University College Cork, Cork, Ireland

**Keywords:** neonatal heart rate, first minute after birth, Doppler ultrasound, auscultation, caesarean section

We read with interest Dr. Hutchon’s commentary on our recent review entitled “Enhanced Monitoring of the Preterm infant during Stabilization in the Delivery Room” (1). We would like to thank him for his kind comments. He raised a number of important points related to one aspect of our review, namely heart rate acquisition in the delivery room. He was disappointed by our “characterization of the usefulness of Doppler ultrasound” in monitoring newborn heart rates (HR) and wondered why we “failed to comment on the impact of delayed cord clamping (DCC) on HR in the first minutes of postnatal life.”

Our review specifically concerned the monitoring of preterm infant stabilization, and we are unaware of any studies, which have assessed the feasibility of Doppler ultrasound monitoring in this population of preterm infants (<32 weeks GA). Doppler ultrasound is certainly a useful method for monitoring newborn infant HR, but it is not commonly practiced, and its use has not been addressed by international guidelines on newborn stabilization (2, 3). Dr. Hutchon has shown that ultrasound HR is readily obtained immediately after birth in a newborn piglet weighing 1 kg (4). He has also shown that Doppler ultrasound can provide immediate heart sounds and that HR can be calculated through a polyethylene wrap from the moment of application in a mannequin model (1). Goenka et al. presented a study at the Pediatric Academic Societies and Asian Society for Pediatric Research meeting in 2014 on Doppler ultrasound in 92 stable infants >35 weeks gestation in the delivery room (5). They found that Doppler ultrasound measurements were feasible in term infants; HR values were comparable to both electrocardiogram (ECG) and pulse oximetry (PO) values; and that these values were available sooner than PO values. The published abstract from this study did not provide details on other important findings, including exact timeframes, in which Doppler ultrasound and ECG values were obtained. We await this detail in a future publication.

Our practice is to have ECG available for HR monitoring, as an adjunct to PO monitoring, for all preterm deliveries <32 weeks gestation (6). This approach is based on a number studies in preterm infants, highlighting the feasibility, accuracy, and availability of HR data within seconds of application (7–10). Simulation training and enhanced teamwork is required to enhance ECG application times, and in our setting, continuous heart rate data are available within seconds of arrival onto the resuscitaire. Based on the current paucity of evidence, it is difficult to comment on the feasibility of HR monitoring using Doppler ultrasound in preterm infants in the DR, and whether there is an added benefit when utilized in conjunction with ECG. Future studies of Doppler certainly seem warranted and should address clinically relevant short-term outcomes. Until these data are available, we cannot make any recommendations on its potential usefulness.

Dr. Hutchon also discusses the impact of DCC on newborn HR, which is an interesting point, and one which deserves increased attention. Guidelines now recommend that for uncompromised term, and preterm infants, DCC should be practiced (2, 3). It is also advised that strategies for providing bedside stabilization during DCC should be explored (11). Dr. Hutchon formed the team, which developed the LifeStart mobile resuscitation trolley (Inditherm, UK, 2013), and stabilization at all gestational ages during DCC is feasible (12). However, we do not know the normal HR range for preterm infants during DCC. Our understanding of newborn HR is derived from the studies of Dawson and colleagues, which were performed in an era when immediate cord clamping was a standard practice (13, 14). As described by Hutchon, Smit et al. have reported HR values obtained by PO (*N* = 109) during DCC, and Katheria et al. have reported values obtained by non-invasive cardiac monitoring (NICOM) (*N* = 20) (15, 16). These studies included term infants only, and one was a feasibility study with small numbers. The studies reported conflicting results, and neither can be extrapolated for preterm infants. Further studies, with adequate study size, are necessary to establish normative HR ranges for preterm infants during DCC. Doppler ultrasound may have a very valuable role in such studies.

Mobile resuscitation trolleys are not equipped with HR monitoring devices, and prospective studies in term and preterm infants are warranted to ascertain which external monitoring device is more feasible in this new stabilization setting and which device is the most appropriate. Once this has been established, larger studies should be performed and normative values obtained. A portable lightweight ultrasound transducer that produces continuous hands-free measurement of HR as described by Hutchon is one such device that deserves further study (1). However, at this current time, we are unsure of the feasibility or appropriateness of placing a large amount of transducer gel onto the thorax of an extremely low birth weight infant and holding the Doppler probe *in situ*. This practice, demonstrated in the accompanying video of Hutchon’s commentary, may be appropriate for term infants, but the device would require modifications to enable hands-free use in preterm infants.

In summary, we wholeheartedly agree that further research is warranted to establish the normative HR range in preterm infants during DCC, and we look forward to further studies that utilize PO, ECG, NICOM, or Doppler ultrasound in this area.

## Author Contributions

DF drafted the initial response. GB, CR, and ED critically revised the manuscript for important intellectual content. All authors agreed on the final manuscript and approved its submission for publication.

## Conflict of Interest Statement

The authors declare that the research was conducted in the absence of any commercial or financial relationships that could be construed as a potential conflict of interest.
